# Supplementary material for: How reliable is the asset score in measuring socioeconomic status? Comparing asset ownership reported by male and female heads of households
Source: PLoS One. 2023 Feb 24;18(2):e0279599. doi: 10.1371/journal.pone.0279599 (PMC9955958; doi:10.1371/journal.pone.0279599)
Supplement: S1 Annex — (DOCX) [file pone.0279599.s001.docx]

**Annex table A1: Reported ownership of different assets by male and female heads of households surveyed in northern Nigeria**

| **Asset type** | **Male-female discrepancies in reporting asset ownership** | | **Percent of households with male-female mismatch in reporting** |
| --- | --- | --- | --- |
|  | **Male head reporting ownership, not female head** | **Female head reporting ownership, not male head** |  |
| Radio | 183 | 120 | 18.3 |
| Television | 92 | 61 | 9.3 |
| Iron | 194 | 115 | 18.7 |
| Fan | 85 | 49 | 8.1 |
| Air Conditioner | 60 | 32 | 5.6 |
| Electric Stove | 58 | 49 | 6.5 |
| Gas Stove | 142 | 94 | 14.3 |
| **Kerosene Lamp** | **241** | **176** | **25.2** |
| Bed | 103 | 43 | 8.8 |
| Mattress | 102 | 63 | 10.0 |
| Mosquito Net | 161 | 157 | 19.2 |
| Refrigerator | 94 | 83 | 10.7 |
| Sewing Machine | 135 | 137 | 16.5 |
| **Table** | **222** | **122** | **20.8** |
| **Sofa** | **255** | **114** | **22.3** |
| **Clock** | **298** | **164** | **27.9** |
| Generator | 126 | 67 | 11.7 |
| Laptop | 47 | 36 | 5.0 |
| Telephone | 45 | 24 | 4.2 |
| **Mobile Phone** | **240** | **188** | **25.9** |
| **Motorcycle** | **233** | **207** | **26.6** |
| **Bicycle** | **201** | **180** | **23.0** |
| Car | 81 | 63 | 8.7 |
| Cart | 118 | 121 | 14.5 |
| Motorboat | 20 | 19 | 2.4 |
| Wheelbarrow | 118 | 135 | 15.3 |
| Plough | 148 | 125 | 16.5 |
| Agricultural Equipment | 196 | 99 | 17.8 |
| Drinking water: Piped into Dwelling | 46 | 16 | 3.8 |
| Drinking water: Piped into yard/plot | 24 | 37 | 3.7 |
| Drinking water: Public Tap | 48 | 43 | 5.5 |
| Drinking water: Tubewell or Borewell | 30 | 78 | 6.5 |
| Drinking water: Protected well | 84 | 81 | 10.0 |
| Drinking water: Unprotected well | 108 | 85 | 11.7 |
| Toilet – Flush latrine (Piped Sewer) | 21 | 16 | 2.2 |
| Toilet – Flush latrine (Septic tank) | 12 | 11 | 1.4 |
| Toilet – Flush latrine (Pit latrine) | 31 | 69 | 6.0 |
| Toilet- Pit latrine | 92 | 174 | 16.1 |
| Toilet- Composting toilet | 246 | 58 | 18.4 |
