# Supplementary material for: How reliable is the asset score in measuring socioeconomic status? Comparing asset ownership reported by male and female heads of households
Source: PLoS One. 2023 Feb 24;18(2):e0279599. doi: 10.1371/journal.pone.0279599 (PMC9955958; doi:10.1371/journal.pone.0279599)
Supplement: Attachment — Submitted filename: Response to reviewers, asset paper, Oct 2022.docx [file pone.0279599.s002.docx]

**Response to reviewers: Asset Score paper, Nigeria**

**Reviewers' comments:**

*Have the authors made all data underlying the findings in their manuscript fully available?*

*The PLOS Data policy requires authors to make all data underlying the findings described in their manuscript fully available without restriction, with rare exception (please refer to the Data Availability Statement in the manuscript PDF file). The data should be provided as part of the manuscript or its supporting information, or deposited to a public repository. For example, in addition to summary statistics, the data points behind means, medians and variance measures should be available. If there are restrictions on publicly sharing data—e.g. participant privacy or use of data from a third party—those must be specified.*

**Response: The data will be available from the first author of the paper. Interested researchers should submit a short research idea for get some idea on how the data will be used. Since the survey had limited number of individuals in some settlements, settlement identifying variables will be removed in addition to other household identifying variables.**

**Reviewer #1 (Professor Shona Kelly):**

*Comment: This is a very interesting paper that provides direct evidence about an assumption made in economics about household asset reporting.*

**Response: Thank you very much Professor Kelly for the positive comment on the overall purpose of the paper. As you have indicated, household asset scores are being used widely by assuming that the reporting of the household assets are correct, unbiased and highly consistent irrespective of the characteristics of the household member reporting the asset ownership.**

*Comment: However, the paper is not structured as expected for a PLOS One paper - the usual paper structure is Introduction, Methods, Results, Discussion, and Conclusion which you follow but the contents in each section are mixed up.*

**Response: Thank you very much for the suggestion. We have reviewed the contents of each of the sections of the paper. Significant revisions have been made to ensure that the contents are relevant for the section. Please see the track changed version showing how the paragraphs were moved from one section to another to ensure relevance.**

*Comments: The following needs to be addressed:*

*1. In an economics paper the introduction can be longer than in a scientific paper but this one is too long and needs to be tightened up considerably, although it is mostly written for a non-economist audience which is good. The methods section contains some background which belongs in the introduction.*

**Response: The paragraph that may be considered “background” information has been moved to introduction. Introduction section was edited to be more concise.**

*Comments: 2. The methods section should stand alone and describe all the work undertaken with the data. You do not describe the various measures in sufficient detail. Do not introduce new information as you write up the results - for example combining urban and semi-urban for some analyses and the calculation of the religiosity score. On page 10, the DHS reference is missing in the references*

**Response: The method section has been rewritten. The DHS reference has been included in the reference list.**

*3. Results. It is usual to start with a descriptive table of the study population. It is difficult to determine when you are talking about Table 1 and Annex Table 1 - try combining the two by eliminating some columns. In Table 5, the number of children may be driven more by the length of time the parents have been together. All the tables need p-values where relevant. Why does the number of assets listed decline steadily for each table? I feel there is too much discussion in this section such as the comments about men knowing about water sources.*

**Response: A simple descriptive statistics table has been added. Annex table 1 is basically a descriptive table reporting ownership of assets and amenities of life and that table has now been moved to the main text with statistical test of differences between reporting of asset ownership by male and female heads. All tables, excepting the new table 4, report all asset types. The focus of table 4 is on the reporting of ownership of assets, not the amenities of life (water source, latrine type used, etc.), which are not assets in the strict sense of the term. Note that, all the assets types, including the amenities of life, were included in the calculation of asset scores.**

*4. Some tables might be clearer as figures. There is some interesting findings here but they are buried in a plethora of analyses. Strip it back to a more straightforward message that is easier to follow. You don't need to report everything you did. I think this paper should just be restricted to the assets analysis while the examination of possible explanatory social factors left for another paper. In my experience with risk perception people's behaviours are not consistent across domains and it is unwise to infer this.*

**Response: Significant editing was carried out to focus on asset scores and male-female differences in the reporting of asset scores. One table has been converted into a graph (figure 1) and included in the manuscript. We have kept some discussions on the potential factors that may influence reporting of the assets to emphasize the point that the outcome is unlikely to be due to random variability in responses and the observed differences may indicate some intrinsic processes in play. Please see the comments of the second reviewer on this. The reviewer has suggested including some potential reasons as well as some discussions on potential alternatives to asset scores.**

**Reviewer #2 (Dr. Klaus Cavalhieri, Ph.D.):**

*Comment: How reliable is the asset score in measuring socioeconomic status? Comparing asset ownership reported by male and female heads of households.*

*The researchers report on a large-scale study, in which over 1,500 households in northern Nigeria were interviewed, to assess whether there are discrepancies in asset scores between male and female heads of household. They report asset scores (derived from ownership of assets and access to important amenities), a common measure to categorize households into different SES groups, are not reliable, as they report significant asset score differences between M and F HH.*

*The authors’ research is novel, and addresses an important topic in the literature, which will be of interest to readership. They report on significant discrepancies on asset scores between male and female heads of households in northern Nigeria, underscoring the low reliability of this commonly used measure of SES classification. The manuscript is well written, and their findings are consistent with their research question and methodological approach.*

**Response: Thank you very much Dr. Cavalhieri for your very positive comments. We also agree that the results directly question the validity and reliability of asset scores as a measure of SES.**

*Comment: One area of concern is in their conclusion - given the low reliability of asset scores, what alternatives are available? I recommend the authors discuss the pitfalls of ‘objective’ measures of SES, and how those might be complemented by ‘subjective’ measures of SES (e.g., subjective social status; one’s perceived position in their own community).*

**Response: This is a very important suggestion. We have discussed potential pitfalls of even the so-called “objective” measures and the importance of complementing the information with subjective measures (based on your suggestion). Concerns with visual verification of reported asset ownership have also been discussed in the manuscript.**

*Comment: Intro -- Discusses issues on variability of income and prestige - could expand on the difficulties in comparing income across populations, further complicating the use of income alone. Making an X amount of money in a rural underserved area allows for significantly different buying power and standard of living in comparison to making the same amount of money in an urban area (where one would likely have less buying power).*

**Response: Limitations on the use of monetary income have been included in the text of the manuscript. Please see last two sentences of first paragraph on page 4 (in the clean version). The sentences incorporated are: Even if we assume that income information can be collected without significant errors, comparing income across population groups and geographic areas is problematic because of price variability and differences in consumption patterns of households. Therefore, income levels alone will not be a good proxy for socioeconomic status of households.**

*Comment: Need more references to support the assertion that ‘surveys on durable asset ownership or access to amenities should be reliable’. Based on which theory? Unless you make your assumptions clear, you are neglecting the significant impact of malingering, and how people place value on different things. The durable assets might not be equally valuable across different people (not only inter-regionally, but interpersonally).*

**Response: This statement is only emphasizing the rationale provided by the proponents of “asset score”-based SES. In the original article in which the asset-based scores were proposed, Filmer et al. argued that the asset-based analysis avoids many of the pitfalls of collecting income and expenditure data, including reliability of the information. In the manuscript, we are simply restating what the proponents of asset-score based analysis suggested. The statement has been slightly modified to indicate that this is the argument offered by the proponents of asset-score based analysis. Please see the first sentence of the last paragraph on page 5 (of clean copy of the manuscript). The reference has also been included (Filmer and Pritchett 2001).**

*Comment: The rationale for the manuscript is clear - and I commend the authors for their clear writing. Nevertheless, the justification for comparing the responses of male and female heads is not well-defined. Why pursue this difference? What could account for possible differences in asset evaluation, that would justify your study? Consider including social determinants that could contribute and help justify your study.*

**Response: The justification is more based on the fact that the current approach of collecting asset information is to interview an adult in the household, implicitly assuming that inter-member variability in reporting assets will be very low, if any. This paper is questioning this implicit assumption.**

*Comment: Method --Were the enumerators different for the male and female heads of household? Were all female heads interviewed by women, and all male heads by men? Consider clarifying this - and whether gender role expectations could have played a part on the data collection (if so, it is important to note this on the method and the limitation section).*

**Response: In method section, we have clarified the survey approach. The enumerators were different for interviewing male and female heads and the interviews were carried out concurrently. Female interviewers interviewed female heads of households and male interviewers interviewed the male heads.**

*Comment: Result -- Males over-reported higher-value items and underreported household items, whereas females over-reported basic amenities. High religiosity and vaccine misinformation associated with higher discrepancy. Table 6 clearly demonstrates the low reliability of asset scores - low reliability for all quintiles (from 40-75%).*

**Response: Thank you very much for a succinct summary of the results.**

*Comments: Discussion/Conclusion --Individual variability in asset scores, particularly as it relates to male and female heads of household. It appears that impression management (attempts to control how the researcher perceives the participants) is interpreted and acted different upon by males and females HH, which is an important finding. However, the authors did not provide clear theoretical interpretations of these findings - they noted that male and female members might have different levels of awareness of assets and amenities, which albeit true, is unlikely to account for over 50% of discrepancy. The authors touch on this already, but it might be relevant to explain why male HH over-report and female HH under-report household resources. What are the differential social, gender, and cultural expectations placed on M and F HH? Why would M be more likely to boast about their assets, and attempt to look better for researchers? Why would F be more likely to display humility and underreport? What is the benefit of this for the participants?*

**Response: We have provided some possible explanations on why male HH tend to inflate their social status. The qualities and roles society expects from male and female heads are quite different and these qualities/roles may affect how they respond to asset ownership questions. Most societies, both in developed and developing economies, consider it very important for a man to be able to financially support a family to be a good husband and father. On the other hand, societies consider honesty, friendly, unselfish, sociable, etc., as important qualities of women, which may explain the display of humility by women when reporting asset ownership.** **We have also cited a research which implies that improving economic status of households tends to benefit male members more than the female members in a traditional society and the benefits of improved economic status often do not translate into significantly lower workload for women. This may imply low propensity of female heads to convey a higher economic status for the household.**

*Comment: Overall, the implications of the study are clear and significant. It is clear that there are issues interpreting asset scores, particularly given their low reliability. Nevertheless - the authors did not address whether subjective assessment of one’s social status and position in their community would be a more reliable way of measuring and classifying participants into social class groups (i.e., socioeconomic groups). Although gender roles and different values placed in assets appear to account for significant differences in asset scores, it is paramount to investigate whether the subjective position in their community would be similar between male and female heads of households. For example, using the MacArthur’s Subjective Social Status Scale, which is a visual representation of one’s community in a ladder, with the ones at the top being the ‘best off’ and the ones at the bottom being the ‘worst off’ - where would male and female HH place themselves? I am aware this was not addressed in this research - but I assume that the subjective evaluation of status would be more reliable between male and female HH, as they compare themselves to their surrounding community (and their nation). This is addressed by the researchers - but there is significant sources of error in using objective measures of SES, given within-group variability in income groups in terms of prestige, buying power, worldview, and standard of living. I highly recommend the authors expand their conclusion - visual verification of asset ownership (as suggested) would still be prone to biases from the researcher and the one reporting (i.e., taking them to verify). Same with education - as a simple example, a university professor in the UK, who usually has a doctorate, typically earns less than a plumber, which does not require advanced education but is a highly skilled and highly paid profession. Education by itself also will not provide an accurate picture of status and SES - a combination of asset scores and subjective social status would likely be better comprehensive picture. Consider including a discussion on subjective social status, and how it can serve as a way to address the reliability issues of asset scores alone.*

**Response: Thank you very much for suggesting this alternative subjective social status scale idea. This is a very important alternative approach that should be considered. We have reviewed several articles using MacArthur’s Subjective Social Status Scale in analysing links to health and wellbeing outcomes. The approach is quite straightforward, easy to implement in filed survey environment and appears to work well in describing underlying differences in health and physical wellbeing. In the manuscript, we have now included this as an alternative approach to asset-scoring. Please see the last paragraph of the manuscript (and a reference to it in the abstract).**
