# Supplementary material for: How reliable is the asset score in measuring socioeconomic status? Comparing asset ownership reported by male and female heads of households
Source: PLoS One. 2023 Feb 24;18(2):e0279599. doi: 10.1371/journal.pone.0279599 (PMC9955958; doi:10.1371/journal.pone.0279599)
Supplement: Attachment — Submitted filename: Responses to reviewer comments second-round reviews.docx [file pone.0279599.s003.docx]

**Responses to reviewer comments**

*We would like to thank Professor Shona Kelly and Dr. Klaus E. Cavalhieri, Ph.D., for taking the time to re-review the manuscript. Reviewing manuscript is a time-consuming activity and our sincere thanks to both of the reviewers.*

*In the paragraphs below, we provide point by point responses to the questions and comments raised in this second-round review. Reviewer’s comments are in bold, responses in italic.*

**1. If the authors have adequately addressed your comments raised in a previous round of review and you feel that this manuscript is now acceptable for publication, you may indicate that here to bypass the “Comments to the Author” section, enter your conflict of interest statement in the “Confidential to Editor” section, and submit your "Accept" recommendation.**

**Reviewer #1: All comments have been addressed**

**Reviewer #2: All comments have been addressed**

*Thank you very much for confirming that we have addressed all the comments you have raised during the first-round of reviews.*

**2. Is the manuscript technically sound, and do the data support the conclusions?**

**The manuscript must describe a technically sound piece of scientific research with data that supports the conclusions. Experiments must have been conducted rigorously, with appropriate controls, replication, and sample sizes. The conclusions must be drawn appropriately based on the data presented.**

**Reviewer #1: Yes**

**Reviewer #2: Yes**

*Again, thank you very much for confirming that the paper represents a technically sound piece of scientific research*

**3. Has the statistical analysis been performed appropriately and rigorously?**

**Reviewer #1: I Don't Know**

**Reviewer #2: Yes**

*We have conducted all statistical analysis with rigor. Appropriate analysis techniques were used in the paper.*

**4. Have the authors made all data underlying the findings in their manuscript fully available?**

**The PLOS Data policy requires authors to make all data underlying the findings described in their manuscript fully available without restriction, with rare exception (please refer to the Data Availability Statement in the manuscript PDF file). The data should be provided as part of the manuscript or its supporting information, or deposited to a public repository. For example, in addition to summary statistics, the data points behind means, medians and variance measures should be available. If there are restrictions on publicly sharing data—e.g. participant privacy or use of data from a third party—those must be specified.**

**Reviewer #1: Yes**

**Reviewer #2: No**

*The data set has been uploaded in STATA format during the submission of first round of review. The data set includes all the variables needed for replication of the study results. Only the personal information or variables that may allow identification of households and household members were excluded from the data set. These variables are not needed for redoing the analysis results presented in the paper.*

**5. Is the manuscript presented in an intelligible fashion and written in standard English?**

**PLOS ONE does not copyedit accepted manuscripts, so the language in submitted articles must be clear, correct, and unambiguous. Any typographical or grammatical errors should be corrected at revision, so please note any specific errors here.**

**Reviewer #1: Yes**

**Reviewer #2: Yes**

*Thank you very much for your evaluation of language aspect of the paper.*

**6. Review Comments to the Author**

**Reviewer #1: Thank you for the changes to the paper. It reads much better now and will be more accessible for non-economists. It is a crucial issue for health inequalities and needs to be disseminated widely in public health and international health circles.**

*Thank you very much for your positive comments on the paper*

**On page six you state “Resorting to strategic responses in reporting asset ownership will be minimal, if any, as the link between asset ownership and means-testing programs is not clear cut.” I think you should be clearer that this is your assumption going in. I’m not sure your findings continue to support this assumption. Especially given your final paragraph about subjective social status. Adler’s ladder has had a considerable amount of subsequent research that refutes its original assumption that the subjective position was based only on the 3 domains listed in the original question wording: income, education and occupational prestige. If you are going to use the ladder choose your anchoring statements carefully – there are quite a few options in the subsequent literature. In reality, there are multiple interacting types of social position that usually are all affecting health at the same time: subjective, objective, and area-based. The correlation between them is surprisingly low.**

*The statement on page six only refers to the rationale provided by researchers who prefer to use “asset-scores” for categorizing households into socioeconomic status groups. This is not the assumption of our paper and the statement has been modified to reflect that this is often an implicit assumption of asset-based scoring approach. In the conclusion, we have suggested the use of Adler’s ladder as a potential alternative. The subjective measure of socioeconomic status is clearly beyond the scope of the paper. Since we are concluding that asset-based scoring is not as reliable as often assumed, we have proposed some alternative approaches. We thank the reviewers for proposing Adler’s ladder as an alternative and the paper only mentions it as a possible subjective approach to consider. If a researcher decides to implement Adler’s ladder, it will be important to determine what anchoring question to use for categorization of households into broad socioeconomic groups.*

**If the “geocodes” for the data was removed, how was urban, rural, and semi-rural determined? England uses population density while other countries use land use measures such as proportion of land under agriculture.**

*The rural-urban is determined by the cluster surveyed and the data set uploaded includes rural-urban categorization for each of the households. Once the households are categorized by rural-urban residences based on cluster number and geocodes, the information is not required for further analysis. In Nigeria, clusters are officially defined as either rural, semi-urban and urban by administrative mechanisms. This study has used administrative definition of rural-urban location.*

**I’m presuming that the majority of the population follows one of the Abrahamic religions so your measure of religiosity is supported as all 3 of those religions encourage people to be honest.**

*The religiosity measure uses three aspects of participation in religious activities and the participation measures should be independent of religious affiliation of the households or household members. In this paper, we are not making any inference about honesty and truthfulness of members belonging to various religious affiliations. The empirical analysis only asked the question whether degree of religiosity (irrespective of religious affiliation) lowers the mis-match between male and female responses.*

**Your discussion seems to be more like further results. Is there no other literature to compare with your findings?**

*We have revised all the three sections: “Results”, “Discussions” and “Conclusions”. Most of the paragraphs in the “Discussion” section have been incorporated into the results section. A major part of the “conclusion” section has been moved into the “discussion” section. The “conclusion” section in the revised version narrowly focuses on policy issues and future research directions.*

**Thank you for the new Table 1. But I calculate the average number of children under 5 Total should be 2.65 rather than 2.85.**

*Thank you for identifying this typographical error. We have corrected the table.*

**People outside of economics will not be familiar with the distinction between assets and amenities. In public health these would not necessarily be distinguished so a few sentences in the methods section would be helpful.**

*In Asset score measurement, access to water, sanitation, etc. was included as part of “asset” owned by the household. Clearly, access to water and sanitation facilities are not assets in the strict sense of the term. To make it clear that not all items in the asset scoring are “assets”, we have explicitly distinguished assets from the amenities of life.*

**Reviewer #2: I believe the authors have adequately addressed all of my concerns from the previous round of reviews. The authors have provided a clear rationale for their methodological decisions, and clearly delineated how they could interpret their results.**

*Thank you very much for your comments and suggestions during the first-round reviews. The comments and suggestions have helped improved this paper significantly.*
